# The rapid inhibition of B-cell activation markers by belimumab was associated with disease control in systemic lupus erythematosus patients

**DOI:** 10.3389/fphar.2023.1080730

**Published:** 2023-02-16

**Authors:** Jing Wang, Bomiao Ju, Li Zhu, Hanchao Li, Jing Luo, Jing Zhang, Nan Hu, Lingfei Mo, Yanhua Wang, Ying Pan, Jing Huang, Xiaohong Lv, Dan Pu, Zhiming Hao, Lan He, Yuanyuan Li

**Affiliations:** Department of Rheumatology and Immunology, First Affiliated Hospital of Xi’an Jiaotong University, Xi’an, Shaanxi, China

**Keywords:** B lymphocytes, belimumab, systemic lupus erythematosus, SYK (spleen tyrosine kinase), Akt

## Abstract

**Objective:** To examine the kinetics of B cell subsets and activation markers in the early stage of belimumab treatment and their correction with treatment response.

**Methods:** We enrolled 27 systemic lupus erythematosus (SLE) patients receiving 6 months belimumab treatment. Flow cytometry was used to test their B cell subsets and activation markers (including CD40, CD80, CD95, CD21^low^, CD22, p-SYK and p-AKT).

**Results:** During belimumab treatment, SLEDAI-2K declined, the proportions of CD19^+^ B cells and naïve B cells decreased, whereas the switched memory B cells and non-switched B cells increased. The larger variations of the B cell subsets and the activation markers were in the first 1 month than the other later time frames. The ratio of p-SYK/p-AKT on non-switched B cell at 1 month was associated with the SLEDAI-2K decline rate in the 6 months of belimumab treatment.

**Conclusion:** B cell hyperactivity was rapidly inhibited in the early stage of belimumab treatment, and the ratio of p-SYK/p-AKT may predict SLEDAI-2K decline.

**Clinical Trial Registration:** https://www.clinicaltrials.gov/ct2/show/NCT04893161?term=NCT04893161&draw=2&rank=1; identifier: NCT04893161.

## 1 Introduction

Systemic lupus erythematosus is a chronic systemic autoimmune disease with heterogeneous clinical manifestations, characterized by B-cell hyperactivity and pathogenic autoantibody formation (Kiriakidou and Ching, 2020). B cells are pivotal to the development of autoantibodies and are a target for intervention in SLE. The agents that target B cells, belimumab and rituximab, are included in the EULAR recommendations as a treatment option for SLE (Fanouriakis et al., 2019). Belimumab is the anti-B-lymphocyte stimulator (BLyS; also known as BAFF) monoclonal antibody and is approved for the treatment of non-renal SLE based on four successful trials (Furie et al., 2011; Navarra et al., 2011; Stohl et al., 2017; Doria et al., 2018) and for the treatment of lupus nephritis (LN) based on the BLISS-LN trial (Furie et al., 2020). However, belimumab is not effective for all patients due to high heterogeneity of SLE, and the response rate of the SLE Responder Index (SRI4) is approximately 50% for the patients with moderate-to-severe SLE (Furie et al., 2011; Navarra et al., 2011; Stohl et al., 2017; Doria et al., 2018). In addition, no definite factor can be used to predict the belimumab response in clinical practice.

B-cell fate determines the progression of SLE. Flow cytometric monitoring of B-cell subsets in the peripheral blood provides a valuable advanced option for monitoring the activity of SLE (Jacobi et al., 2003). During belimumab treatment for SLE patients, circulating concentrations of all B-cell types [including CD19^+^ B cells, CD20^+^ B cells, naïve B cells (CD19^+^, CD20^+^, and CD27^−^), activated B cells (CD20^+^ and CD69^+^), plasmacytoid B cells (CD19^+^, CD20^+^, and CD138^+^), SLE subset plasma cells (CD19^+^, CD27^BRIGHT+^, and CD38^BRIGHT+^), short-lived plasma B cells (CD19^+^,CD20^−^, and CD27^BRIGHT+^), and plasma B cells (CD19^+^, CD20^−^, and CD138^+^)], except memory B cells (CD19^+^, CD20^+^, and CD27^+^), decreased rapidly in the first 24 weeks and then declined slowly through to week 72, and the decline in numbers of most B-cell subsets (naïve, activated, plasmacytoid, and plasma B cells) had plateaued by weeks 288 or 312 (9). Similar results were observed in the BLISS-52, BLISS-76, and BLISS-76 continuation studies (Stohl et al., 2012; Furie et al., 2018). Memory B cells increased at 8 weeks, 12 weeks, and 24 weeks of belimumab treatment and then subsequently declined progressively over time (Jacobi et al., 2010; Stohl et al., 2012; Struemper et al., 2022). B-cell homeostasis was strikingly disturbed in SLE patients (Zhu et al., 2018); the agents that target B cells tended to restore B-cell homeostasis (Anolik et al., 2004); the significant variation in B cells occurred in the early rituximab treatment (4 weeks and 8 weeks) and was associated with SLE relapse (Lazarus et al., 2012). The dynamic change in B-cell subset frequencies in the early stage of belimumab treatment was speculated to be associated with SLE remission. Nonetheless, no study focused on the change in B-cell subsets in early belimumab treatment and its correlation with the efficacy. The proportion of B-cell subsets cannot completely reflect the B-cell hyperactivity state, and the kinetics of B-cell activation markers are still unclear during belimumab treatment.

In the early stage of belimumab treatment, the dynamic change in B-cell subset frequencies and B-cell activation markers was delineated by comprehensive flow cytometric analysis, and the correlation was analyzed between B cells and clinical outcomes following belimumab treatment, in order to figure out the potential marker to predict the belimumab response.

## 2 Materials and methods

### 2.1 Participants

This study was a prospective, single-center cohort study (ClinicalTrials.gov identifier: NCT04893161). From December 2020 to January 2022, SLE patients who received belimumab treatment were recruited from the Department of Rheumatology and Immunology, The First Affiliated Hospital of Xi’an Jiaotong University, Shaanxi, China, according to the following inclusion criteria: (i) patients with age more than 18 years and (ii) patients fulfilled the Systemic Lupus International Collaborating Clinics (SLICC) 2012 criteria (Petri et al., 2012). Patients who had definitely diagnosed infection, cancer, or end-stage diseases were excluded from the study. This study was conducted in accordance with the Declaration of Helsinki, and the protocol was approved by the Ethics Committee of The First Affiliated Hospital, School of Medicine, Xi’an Jiaotong University (no. XJTU1AF2020LSK-278).

### 2.2 Treatment and follow-up

All patients with SLE were administered 10 mg/kg intravenous infusion of belimumab for over 1 h on days 0, 14, and 28 and every 28 days for 6 months. The standard care treatment of SLE was permitted. The regimen of standard care was based on the SLE manifestations of patients and the judgment of the doctors. All patients are evaluated at months 0 (T0), 1 (T1), 3 (T3), and 6 (T6).

**FIGURE 1:**
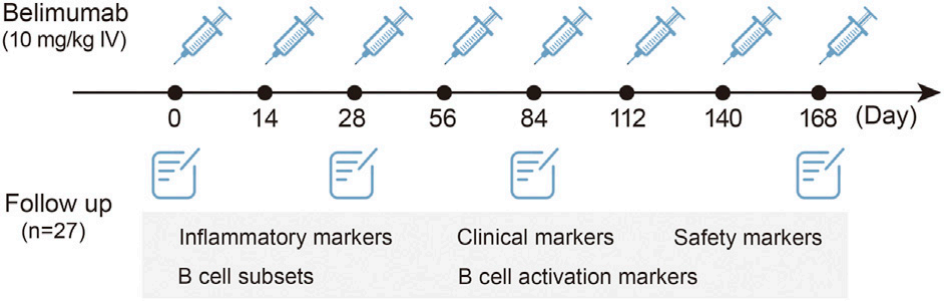
Flow chart of the study.

The demographic and clinical features were obtained at baseline, and clinical symptoms, laboratory data, medications, and SLE Disease Activity Index 2000 (SLEDAI-2K) (Gladman et al., 2002) were ascertained at each follow-up point. The laboratory data included anti-dsDNA, C-reactive protein (CRP), erythrocyte sedimentation rate (ESR), lgG, complements C3 and C4, 24-h urine protein, tumor necrosis factor-α (TNF-α), interferon-α (IFN-α), IFN-γ, interleukin-1β (IL-1β), IL-2, IL-4, IL-5, IL-6, and IL-17. The flow chart is shown in Figure 1.

The primary outcome was dynamical changes in B-cell subset proportions and B-cell activation markers during belimumab treatment. The secondary outcomes were as follows: the correlation between B cells (including subset proportions and activation markers) and disease markers (including SLEDAI-2K decline rate, clinical markers, and the dynamic changes in SLEDAI-2K, anti-dsDNA, prednisone dose, IgG, ESR, CRP, C3, C4, ALB, and 24-h urine protein); and the dynamic changes in inflammation cytokines during the follow-up. The definition of the SLEDAI-2K decline rate was given as follows: (SLEDAI-2K-T6 minus SLEDAI-2K-T0)/SLEDAI-2K-T0.

### 2.3 Laboratory methods

Venous blood was collected from the 27 SLE patients. The tests (including anti-dsDNA, lgG, C3, C4, CRP, ESR, ALB, 24-h urine protein, TNF-α, IFN-α, IFN-γ, IL-1β, IL-2, IL-4, IL-5, IL-6, and IL-17) were performed in the central laboratories of The First Affiliated Hospital, School of Medicine, Xi’an Jiaotong University. The aforementioned cytokines were tested using cytometric bead array (CBA) (Medeiros and Gomes, 2019).

Flow cytometry was used to test lymphocyte immunophenotyping. Peripheral blood mononuclear cells (PBMCs) were isolated by Ficoll gradient centrifugation (Tianjing Haoyang Biological Company, China) and then washed and resuspended in PBS and 1% BSA to obtain the cell suspension of 1 × 10^6^ cells/100 µL. All samples were stained using APC-labeled anti-CD19, FITC-labeled anti-lgD, and PE-labeled anti-CD27 (BD Biosciences, San Diego, CA, United States) for B cells. B-cell activation markers (including CD40, CD80, CD95, CD21^low^, and CD22) were tested in 12 patients. After staining with antibodies, cells were assessed using CytoFLEX (Beckman Coulter, United States), and the data analysis was performed using CytExpert.

For B-cell *in vitro* activation, PBMCs (10^7^/ml) were incubated for 30 min at 37°C and then stained using APC-labeled anti-CD19, FITC-labeled anti-lgD, and PE-labeled anti-CD27 for 30 min at 37°C. Next, the PBMCs were stimulated for 5 min with F(ab’)2 anti-IgM (2 μg/mL) and fixed with an equal volume of 100 μL of BD Cytofix Fixation Buffer (catalog no. 554655; BD Biosciences) for 10 min at 4°C. After fixation, the PBMCs were centrifuged at 400 ×g for 3 min, followed by two consecutive washes with MACS buffer, and then stained with a staining mix containing mAbs to markers, phosphorylated spleen tyrosine kinase (p-SYK) and p-AKT, for 1.5 h at 4°C in the dark. Subsequently, the PMBCs were washed with MACS buffer and resuspended in a volume of 150 μL of MACS buffer for testing on the flow cytometer.

### 2.4 Statistical analysis

The analysis was performed using SPSS software 13.0 (SPSS Inc., Chicago, IL, United States). Descriptive analysis (calculations of averages, proportions, or rates) was conducted. The Shapiro–Wilk test and Levene’s statistics were used to evaluate the normality and homogeneity of the variance, respectively. According to the situation, the significance of mean differences between the two groups was assessed by Student’s t-test or the Mann–Whitney *U*-test, and the differences in correlation were assessed by Pearson’s or Spearman’s analysis. Univariate logistic regression analysis was used to determine odds ratios (ORs) and their 95% confidence intervals (CIs) of the variables. *p-*values less than 0.05 were considered significant.

## 3 Results

### 3.1 Baseline characteristics and clinical efficacy of SLE patients treated with belimumab

A total of 27 SLE patients receiving belimumab treatment were enrolled in the current study and followed up for 6 months. Peripheral B-cell subsets were tested dynamically for all the patients. The B-cell activation markers were tested for 12 patients among them. Demographic and baseline characteristics of these patients are shown in Table 1. The mean age was 32.81 ± 11.33 years. The proportion of female patients was 96.30% (26/27). The mean disease duration was 7.04 ± 6.36 years. The mean SLEDAI-2K score was 9.00 ± 5.06 at baseline. No significant differences were found between the whole group of patients and the subgroup of patients tested for the B-cell activation markers (Table 1).

**TABLE 1:** Demographic and baseline characteristics of SLE patients.

Parameter	Total number of patients (N = 27)	Number of patients detected with B-cell activation regulators (N = 12)	*p*-value
Age, years^a^	32.81 ± 11.33	29.83 ± 8.81	0.521
Gender, female (%)	26 (96.30)	11 (91.67)	0.526
Duration of disease, years^a^	7.04 ± 6.35	3.80 ± 6.06	0.074
SLEDAI-2K score^a^	9.00 ± 5.06	10.42 ± 4.29	0.405
≤6	11 (40.74)	2 (16.67)	0.311
7–12	10 (37.04)	7 (58.33)	
>12	6 (22.22)	3 (25.00)	
Dominant organ involvement (%)			
Mucocutaneous	16 (59.26)	7 (58.33)	0.985
Musculoskeletal	14 (51.85)	5 (41.67)	
Hematology	10 (37.04)	4 (33.33)	
Vasculitis	4 (14.81)	1 (8.33)	
Renal	17 (62.96)	7 (58.33)	
Cardiovascular/respiratory	1 (3.70)	0	
Medication (%)			
Glucocorticoid	12.59 ± 9.59	14.17 ± 9.73	0.388
Prednisone or equivalent >7.5 mg/d	16 (59.3)	10 (83.33)	0.269
Mycophenolate mofetil	14 (51.85)	8 (66.67)	0.389
Tacrolimus	10 (37.04)	4 (14.81)	1.000
Cyclosporin A	1 (3.70)	0	1.000
Leflunomide	1 (3.70)	0	1.000
Azathioprine	2 (7.41)	0	1.000
Hydroxychloroquine	22 (81.48)	10 (83.33)	1.000
Biomarker			
Proteinuria, g^a^	0.79 ± 1.26	1.03 ± 1.51	0.636
>0.5 g/24 h	11 (40.74)	6 (50.00)	0.590
Anti-dsDNA, IU/mL^a^	57.22 ± 41.25	63.96 ± 41.64	0.647
>7 IU/ml (%)	23 (85.19)	11 (91.67)	1.000
Serum IgG, g/L^a^	14.58 ± 7.93	10.80 ± 4.32	0.107
>16 g/L	7 (25.93)	2 (16.67)	0.693
Complement C3, g/L^a^	0.70 ± 0.23	0.72 ± 0.23	0.807
<0.8 g/L	20 (74.07)	8 (66.67)	0.709
Complement C4, g/L^a^	0.13 ± 0.07	0.14 ± 0.07	0.723
<0.1 g/L	12 (44.4)	4 (33.33)	0.729

^a^ The values are expressed as the mean ± standard deviation. Abbreviations: N, number; SLEDAI-2K, SLE Disease Activity Index 2000.

**FIGURE 2:**
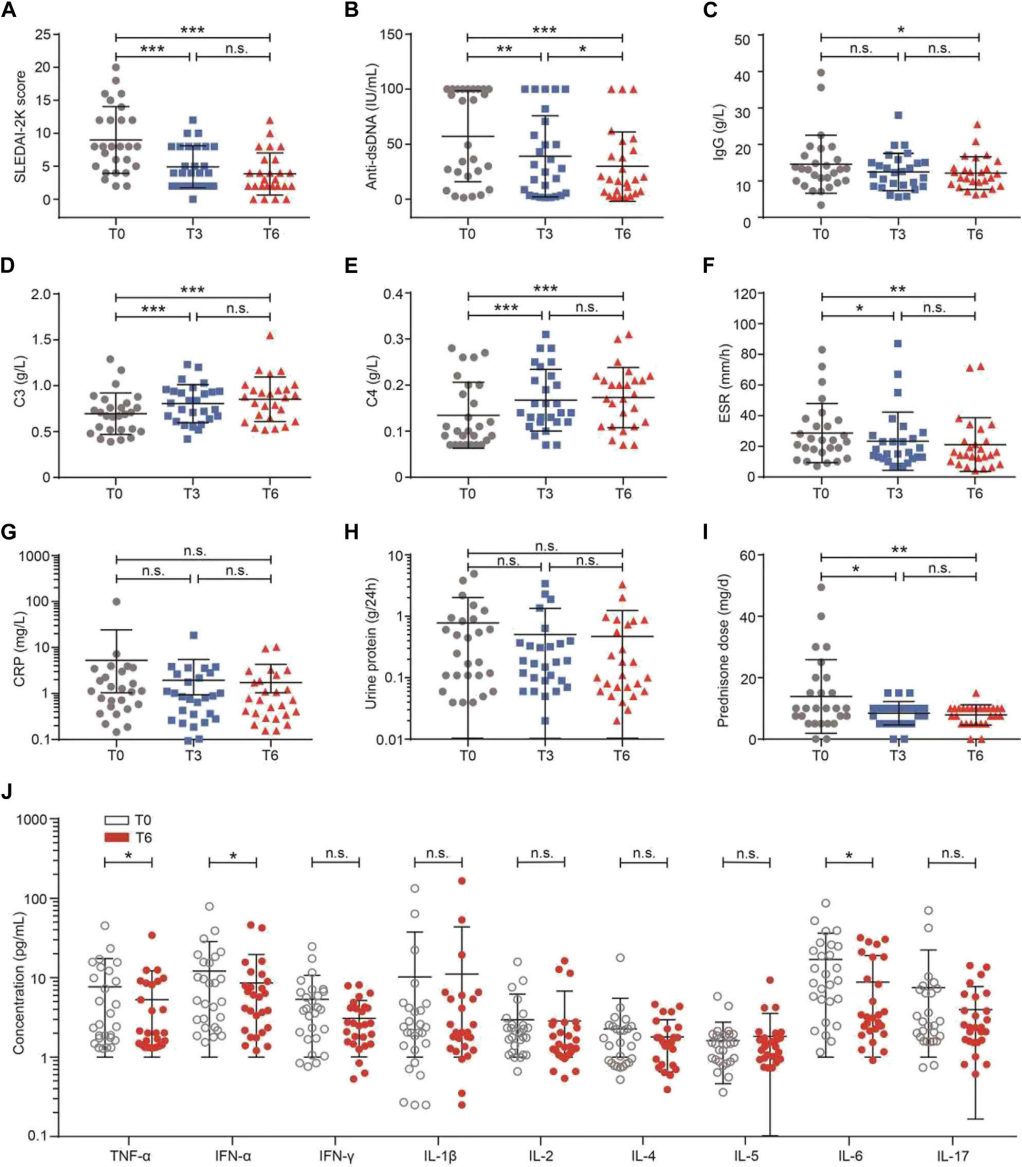
Effects of belimumab treatment on clinical markers in the patients with SLE. **(A–J)** Change in the SLEDAI-2K score, anti-dsDNA, IgG, complement C3, complement C4, ESR, CRP, 24-h urine protein, dose of prednisone, and levels of inflammation cytokines. Abbreviations: CRP, C-reactive protein; DN, double-negative; ESR, erythrocyte sedimentation rate; SLE, systemic lupus erythematosus; SLEDAI-2K, SLE Disease Activity Index 2000; T, time.

As shown in Figures 2A–I, there was a significant decline in SLEDAI-2K (T0 vs T6: 9.00 ± 5.06 vs 3.85 ± 3.18, *p* < 0.001) during belimumab treatment. The mean of the decreased SLEDAI-2K score was 5.22 ± 4.56. At T6, 81.48% (22/27) of patients had a SLEDAI-2K score ≤6 and 18.52% (5/27) of patients had a SLEDAI-2K score between 7 and 12. A decline was observed in the level of anti-dsDNA (T0 vs T6: 57.22 ± 41.25 vs 29.88 ± 31.31 IU/ml, *p* = 0.025), and a rise was observed in complements C3 (T0 vs T6: 0.70 ± 0.23 vs 0.85 ± 0.24 g/L, *p* = 0.011) and C4 (T0 vs T6: 0.13 ± 0.07 vs 0.17 ± 0.07 g/L, *p* = 0.027). ESR (T0 vs T6: 29.30 ± 20.50 vs 20.88 ± 17.56 mm/h, *p* = 0.054), CRP (T0 vs T6: 5.09 ± 18.44 vs 1.66 ± 2.50 mg/L, *p* = 0.346), IgG (T0 vs T6: 14.58 ± 7.93 vs 12.10 ± 4.60 mg/L, *p* = 0.203), proteinuria levels (T0 vs T6: 0.79 ± 1.26 vs 0.50 ± 0.80 g/24 h, *p* = 0.317), and the dose of prednisone (T0 vs T6: 13.89 ± 12.00 vs 7.87 ± 3.30 mg/d, *p* = 0.109) tended to decrease, although there was no significant difference.

The levels of nine serum cytokines were compared between baseline and 6 months of belimumab treatment. As shown in Figure 2J, the levels of TNF-α (T0 vs T6: 7.66 ± 9.71 vs 5.27 ± 6.89 IU/ml, *p* = 0.047), IFN-α (T0 vs T6: 5.35 ± 5.38 vs 3.09 ± 2.08 IU/ml, *p* = 0.037), and IL-6 (T0 vs T6: 17.00 ± 19.21 vs 8.79 ± 10.27 IU/ml, *p* = 0.031) were lower after 6 months of treatment than baseline. There was no difference in IFN-γ (*p* = 0.235), IL-1β (*p* = 0.507), IL-2 (*p* = 0.796), IL-4 (*p* = 0.492), IL-5 (*p* = 0.208), and IL-17 (*p* = 0.171, Supplementary Figure S1).

### 3.2 Dynamical changes in B-cell subset proportions and B-cell activation markers during belimumab treatment

**FIGURE 3:**
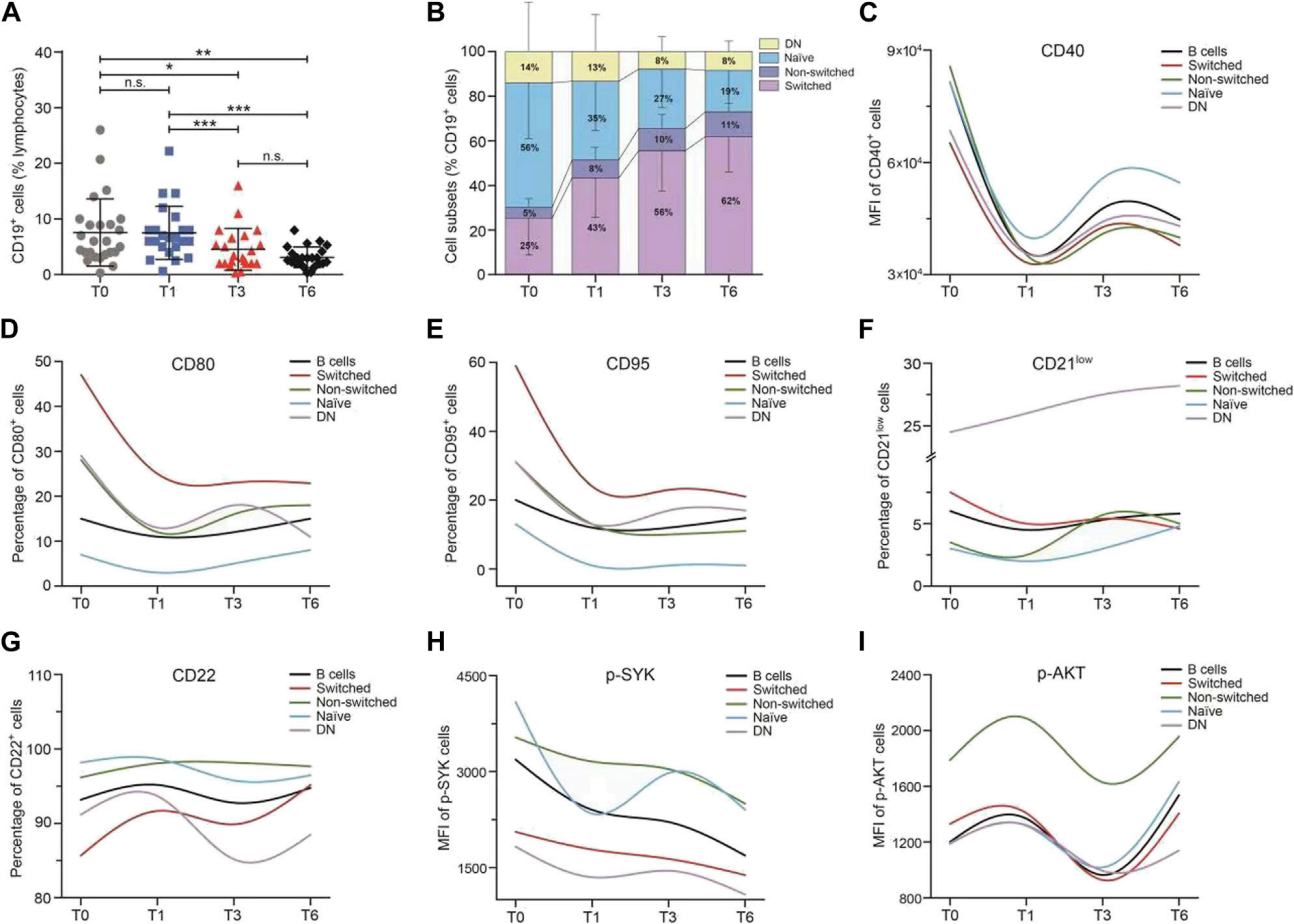
Median change from baseline over time for B-cell subsets and B-cell activation regulators in the SLE patients during belimumab treatment. **(A–I)** Change in the percentage of CD19^+^ B cells, frequencies of B-cell subsets, MFI of CD40, percentage of CD80, CD95, CD21^low^, and CD22, and MFI of p-SYK and p-AKT. Abbreviations: DN, double-negative; SLE, systemic lupus erythematosus; T, time.

During 6 months of continued belimumab treatment, circulating CD19^+^ B cells declined progressively over time (T0 vs T3: 7.57 ± 6.03 vs 4.55 ± 3.76 IU/ml, *p* = 0.032; T0 vs T6: 7.57 ± 6.03 vs 3.12 ± 1.85 IU/ml, *p* < 0.001, Figure 3A). CD27 and IgD were used to identify four differentiation subsets of CD19^+^ B cells. As shown in Figure 3B, the proportions of switched memory B cells (*p* < 0.001) and non-switched B cells (*p* = 0.007) increased, and the proportions of naïve B cells (*p* < 0.001) and DN B cells decreased, although there was no statistical difference observed in DN B cells (*p* = 0.368). The larger variations were observed from baseline to 1 month in switched (T0 vs T1: 25.20 ± 16.52 vs 43.03 ± 17.59 IU/ml, *p* = 0.001), non-switched (T0 vs T1: 4.78 ± 3.87 vs 8.05 ± 5.65 IU/ml, *p* = 0.022), and naïve memory B cells (T0 vs T1: 55.67 ± 24.92 vs 35.02 ± 21.87 IU/ml, *p* = 0.003) than the later time frames.

As shown in Figures 3C–I, the variation trend of each B-cell activation marker (including CD40, CD80, CD95, CD21^low^, CD22, p-SYK, and p-AKT) expression was analogous among the CD19^+^ B cells and four memory B-cell subsets. The levels of CD40, CD80, CD95, CD21^low^, and p-SYK decreased rapidly in the first month and then increased slowly through to 6 months. On the contrary, the levels of CD22 and p-AKT increased in the first month and then declined. The larger variations in B-cell activation regulators were also observed from baseline to 1 month than the later time frames. The expression of CD40 and CD22 was higher in naïve B cells than the other memory B-cell subsets. The switched B cells had the highest expression of CD80 and CD95 and the non-switched B cells had the highest expression of p-SYK and p-AKT, among the B-cell subsets. It was further analyzed for ascertaining the correlation between the change in B-cell subset frequencies, the B-cell activation markers at an early stage, and the SLE disease activity markers (including SLEDAI-2K, anti-dsDNA, IgG, C3, C4, proteinuria, ESR, and CRP), and no correlation was found.

### 3.3 p-SYK/p-AKT at 1 month was associated with the SLEDAI-2K decline rate

As shown previously, a decrease was observed in SLEDAI-2K after 6 months of belimumab treatment. As mentioned in Materials and Methods, the definition of the SLEDAI-2K decline rate was given as (SLEDAI-2K-T6 minus SLEDAI-2K-T0)/SLEDAI-2K-T0. The median of the SLEDAI-2K decline rate was -0.67. The patients were divided into two groups based on whether greater than or less than the median of the SLEDAI-2K decline rate. Then, the correlation between B cells and the SLEDAI-2K decline rate was analyzed by univariate logistic regression analysis. Each B-cell subset proportions and their activation markers had no association with the SLEDAI-2K decline rate at any time points (Supplementary Table S1). Considering the inverse change trend of p-SYK and p-AKT during belimumab treatment, the ratio of p-SYK/p-AKT was further analyzed, and the data showed that the ratio of p-SYK/p-AKT on non-switched B cells at 1 month was associated with the SLEDAI-2K decline rate [HR (95%CI): 34.373 (1.113, 1061), *p* = 0.043, Table 2].

**TABLE 2:** Association between the ratio of p-SYK/p-AKT at 1 month and SLEDAI-2K decline rate by univariate analysis in the SLE patients.

Variable	Univariate OR (95%CI)	*p*-value
p-SYK/p-AKT on B cells at T0	0.840 (0.328, 2.151)	0.716
p-SYK/p-AKT on B cells at T1	170.995 (0.403, 72,630)	0.096
p-SYK/p-AKT on B cells at T3	62.231 (0.117, 32,970)	0.197
p-SYK/p-AKT on B cells at T6	0.156 (0.002, 10.189)	0.384
p-SYK/p-AKT on switched B cells at T0	0.976 (0.253, 3.765)	0.972
p-SYK/p-AKT on switched B cells at T1	159.166 (0.340, 74,420)	0.106
p-SYK/p-AKT on switched B cells at T3	94.370 (0.080, 110,700)	0.207
p-SYK/p-AKT on switched B cells at T6	1.934 (0.079, 47.475)	0.686
p-SYK/p-AKT on non-switched B cells at T0	0.862 (0.285, 2.604)	0.792
p-SYK/p-AKT on non-switched B cells at T1	34.373 (1.113, 1061)	**0.043**
p-SYK/p-AKT on non-switched B cells at T3	2.500 (0.407, 15.341)	0.322
p-SYK/p-AKT on non-switched B cells at T6	0.495 (0.034, 7.210)	0.607
p-SYK/p-AKT on naïve B cells at T0	0.887 (0.381, 2.061)	0.780
p-SYK/p-AKT on naïve B cells at T1	0.979 (0.116, 8.295)	0.985
p-SYK/p-AKT on naïve B cells at T3	2.317 (0.348, 15.421)	0.385
p-SYK/p-AKT on naïve B cells at T6	0.652 (0.056, 7.540)	0.732
p-SYK/p-AKT on DN B cells at T0	0.849 (0.226, 3.187)	0.809
p-SYK/p-AKT on DN B cells at T1	270.415 (0.037, 1,963,000)	0.217
p-SYK/p-AKT on DN B cells at T3	4.164 (0.057, 305.497)	0.515
p-SYK/p-AKT on DN B cells at T6	0.018 (0, 30.118)	0.288

Abbreviations: CI, confidence interval; DN, double-negative; OR, odds ratio; SLE, systemic lupus erythematosus; T, time. The bold value was less than 0.05 and considered significant.

### 3.4 The correlation between B cells and IgG, anti-dsDNA, and inflammation cytokines

**FIGURE 4:**
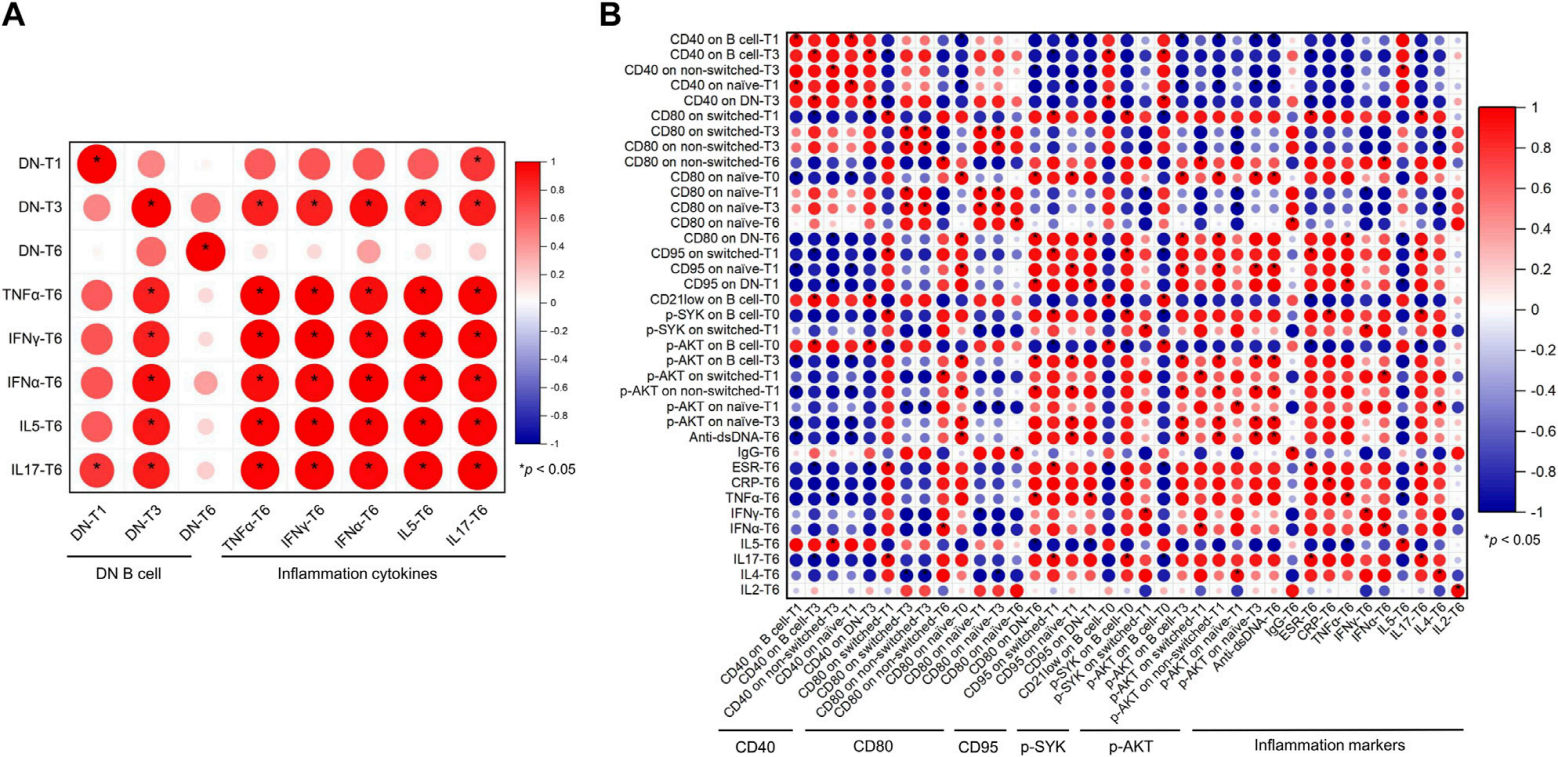
Correlation between B cells and clinical markers in the SLE patients. **(A)** Correlation between DN B cells and inflammation cytokines. **(B)** Correlation between B-cell activation regulators and anti-dsDNA, IgG, and inflammation markers. Abbreviations: CRP, C-reactive protein; DN, double-negative; ESR, erythrocyte sedimentation rate; IL, interleukin; IFN, interferon; SLE, systemic lupus erythematosus; T, time; TNF, tumor necrosis factor.

B cells play main roles in IgG secretion, antigen presentation, and inflammation cytokine production. The correlation between B cells and IgG, anti-dsDNA, and inflammation markers at T6 was further analyzed in these SLE patients. First, the proportion of DN B cells (T1, T3, and T6) was positively associated with inflammation cytokines (IL-5, IL-17, TNF-α, IFN-α, and IFN-γ, Figure 4A).

Second, B-cell activation markers were further analyzed. As shown in Figure 4B, anti-dsDNA was positively associated with CD80 on naïve cells (T0), CD95 on naïve cells (T1), and p-AKT on B cells (T0), non-switched cells (T1), and naïve (T3) B cells. IgG was positively correlated with CD80 on naïve B cells (T6). It was complicate to ascertain the correlation between B-cell activation regulators and inflammation markers. p-SYK on B cells (T0) and switched B cells (T1) was positively correlated with CRP, IFN-γ, and IL-17. p-AKT on B cells (T3) and naïve B cells (T1) was positively correlated with IFN-α and IL-4, and p-AKT on B cells (T0) was negatively associated with ESR and IL-17. CD21^low^ on B cells (T0) was negatively correlated with ESR. CD80 and CD95 were mainly positively associated with ESR, TNF-α, IFN-α, and IL-17 and negatively correlated with IL-4, IL-5, and IFN-γ.

## 4 Discussion

Disturbed B-cell homeostasis was observed in SLE, which included an increased frequency of DN memory B cells and declined non-switched B cells (Zhu et al., 2018). Belimumab treatment facilitated the reestablishment of B-cell homeostasis (Wallace et al., 2009; Stohl et al., 2012; Furie et al., 2018; Struemper et al., 2022). In the current study, the proportions of B-cell subsets changed over time, and the levels of B-cell activation markers significantly varied in the first month during belimumab treatment. p-AKT on non-switched cells at 1 month was positively correlated with anti-dsDNA at 6 months, and the ratio of p-SYK/p-AKT on non-switched B cells at 1 month was associated with the SLEDAI-2K decline rate during 6 months of belimumab treatment, which may become a potential marker to predict belimumab treatment.

It contributes to the development of SLE for the alterations of memory B-cell subsets and activation state. Our previous study identified the proportion of B-cell subsets in 130 SLE patients and 55 healthy controls, and the frequency of non-switched memory cells reduced in the SLE patients compared with the healthy controls and more significantly decreased in the patients with a long-term disease duration than the new-onset SLE patients (Zhu et al., 2018), which may be partly due to non-switched B-cell accumulation in the germinal center and is responsible for persistent autoantibody production in SLE patients (Rodriguez-Bayona et al., 2010; van Zelm, 2012; Malkiel et al., 2016). During belimumab treatment, the proportions of non-switched B cells increased, and the larger variations were observed from baseline to 1 month than the later time frames. In the context of SLE, non-switched B cells were able to recruit into lymphoid tissues, reinitiate the germinal center reaction, and proliferate and differentiate into mature effector cells during secondary auto-antigen exposure (Seifert and Kuppers, 2009). The peripheral non-switched B cell increase during belimumab treatment may reflect the control of autoantibody secretion and disease severity in SLE patients to a certain extent. On the other hand, we observed the suppression of the B-cell hyperactivity state induced by belimumab treatment. CD40 and CD80 have been shown to play important roles in T-cell-mediated B-cell activation, including stimulation and co-stimulation of cell growth, switch recombination and transcriptional regulation (Bishop and Hostager, 2001; Guo et al., 2021), regulation of cell death, and rescuing B cells from anti-Ig-induced apoptosis (Suvas et al., 2002). CD95-mediated death plays an essential role in maintaining B-cell tolerance (Huck et al., 1998), and CD95, as evidence suggested, may be a useful marker to identify memory B cells with an activated phenotype and a biomarker for lupus activity (Jacobi et al., 2008). An expansion of CD21^low^ B cells was observed in SLE(29), which displayed a potential role as antigen-presenting cells, when enriched for autoreactive B-cell receptors (BCRs), potentially contributing to autoimmunity reactions as cognate interaction partners of autoreactive T cells at sites of inflammation (Vale and Schroeder, 2010). We observed that the levels of CD40, CD80, CD95, and CD21^low^ decreased rapidly in the first month and then increased slowly through to 6 months of belimumab treatment, which indicated the rapid inhibition of B-cell activation in the early stage of belimumab treatment and B-cell homeostasis reestablishment during the later time of treatment.

The innate-like B cells (including B1 cells and marginal-zone B cells) with autoreactive B-cell receptor (BCR) expression contributed to the development of SLE, especially in the autoantibody production and inflammation cytokine secretion (Carnrot et al., 2011; Vinuesa and Chang, 2013; Ma et al., 2019). Non-switched B cells displayed a marginal-zone B-cell phenotype (van Zelm, 2012). The BCR activation state in the non-switched B cells was associated with SLE disease activity. In the current study, p-AKT on non-switched B cells at 1 month was positively correlated with anti-dsDNA levels at 6 months of belimumab treatment (Figure 4B). The dynamic change in p-SYK indicated an inverse trend compared to that in p-AKT levels during belimumab treatment (Figures 3H,I). Our further analysis showed the ratio of p-SYK/p-AKT on non-switched B cells at 1 month was associated with the SLEDAI-2K decline rate (Table 2). BLyS interacted with the TNF receptor family member, BAFFR, providing mature B cells with pro-survival signals though not exclusively but *via* a SYK-dependent signaling activation (Schweighoffer et al., 2013). In addition, CD19 drove NF-κB/AKT activation to maintain mature B-cell survival, not involving SYK (Hobeika et al., 2015). Belimumab inhibited the activity of soluble BLyS (Cancro et al., 2009) and facilitated autoimmune B-cell apoptosis by suppression of the BAFFR-SYK signal in a partially selective manner (Petri et al., 2008), whereas belimumab had a relatively indirect impact on the CD19-NF-κB/AKT signal, which may partly explain the opposite change trend of p-SYK and p-AKT levels during belimumab treatment and the association between the ratio of p-SYK/p-AKT and the SLEDAI-2K decline rate. SYK and AKT were much closer to BCR signaling, and the change in the BCR signaling induced by belimumab can blunt B-cell response to the pathogens. The infection was the important adverse effect of belimumab treatment. In this study, patients had no infection during the treatment.

In SLE, CD21^low^ B cells and DN memory B cells expanded (Zhu et al., 2018; Freudenhammer et al., 2020) and were described as age-associated B cells with proinflammatory characteristics (Claes et al., 2016). DN B cells showed a proinflammatory cytokine profile, including IL-5, IL-17, TNF-α, IFN-α, and IFN-γ (Figure 4A), which could contribute to SLE pathology. DN B cells showed the highest expression of CD21^low^ among B-cell subsets (Figure 3F). In consistent with previous studies, DN B cells, as age-associated B cells, were related to the typical inflammatory microenvironment, characterized by a general increase in proinflammatory cytokines, and were associated with increased disease-specific autoantibodies in SLE (Rodriguez-Bayona et al., 2010; van Zelm, 2012; Malkiel et al., 2016, Wei et al., 2007). During belimumab treatment, the proportions of DN B cells showed a decline trend, although there was no statistical difference. Furthermore, CD21^low^ expression progressively increased in DN B cells. This suggested the risk of SLE flare after belimumab withdrawal. During belimumab treatment, the levels of TNF-α, IFN-α, and IL-6 decreased (Figure 2J). First, in the context of SLE, B cells can respond to nucleic acid material through direct antigen recognition and surface IgM receptors in the microenvironment containing type Ⅰ IFN and other cytokines. Once activated, these autoreactive B cells bypass tolerance checkpoints, mature, expand, and secrete inflammation cytokines. Second, B cells, as antigen-presenting cells, can present auto-antigen to T cells and promote inflammation cytokine secretion (Yap and Chan, 2019). In the process of belimumab treatment, B cells, especially autoreactive B cells, were deleted, so the levels of the aforementioned inflammation cytokines decreased.

This is the first report that shows the detailed profile of B-cell subsets and B-cell activation markers in the early stage of belimumab treatment. Although the numbers of patients were moderate and there were no data on long-term belimumab treatment, a prospective cohort study with a large sample size is necessary to evaluate the relationship between B-cell subsets and SLE disease control.

In conclusion, B-cell hyperactivity was rapidly inhibited in the early stage of belimumab treatment, and the ratio of p-SYK/p-AKT on non-switched B cells at 1 month was associated with the SLEDAI-2K decline rate, which may be a potential marker to predict the belimumab treatment response.

## Data Availability

The raw data supporting the conclusion of this article will be made available by the authors, without undue reservation.
