# Supplementary material for: The rapid inhibition of B-cell activation markers by belimumab was associated with disease control in systemic lupus erythematosus patients
Source: Front Pharmacol. 2023 Feb 16;14:1080730. doi: 10.3389/fphar.2023.1080730 (PMC9978353; doi:10.3389/fphar.2023.1080730)
Supplement: Supplementary file 1 [file Table1.DOCX]

**Table S1.** No correlation between memory B cell subsets frequency, their activation markers and SLEDAI-2K reduction rate by univariate analysis in the SLE patients

| Variable | Univariate OR (95% CI) | *P* value |
| --- | --- | --- |
| Switched B cell at T0 | 1.013 (0.965, 1.064) | 0.601 |
| Switched B cell at T1 | 0.986 (0.940, 1.035) | 0.570 |
| Switched B cell at T3 | 1.004 (0.958, 1.053) | 0.864 |
| Switched B cell at T6 | 1.043 (0.981, 1.108) | 0.176 |
| Non-switched B cell at T0 | 1.067 (0.862, 1.320) | 0.553 |
| Non-switched B cell at T1 | 0.980 (0.844, 1.137) | 0.787 |
| Non-switched B cell at T3 | 0.964 (0.837, 1.111) | 0.613 |
| Non-switched B cell at T6 | 0.928 (0.807, 1.066) | 0.292 |
| Naïve B cell at T0 | 0.991 (0.959, 1.024) | 0.593 |
| Naïve B cell at T1 | 1.009 (0.970, 1.049) | 0.666 |
| Naïve B cell at T3 | 0.993 (0.945, 1.043) | 0.774 |
| Naïve B cell at T6 | 0.973 (0.917, 1.033) | 0.376 |
| DN B cell at T0 | 1.002 (0.966, 1.039) | 0.907 |
| DN B cell at T1 | 1.002 (0.951, 1.055) | 0.949 |
| DN B cell at T3 | 1.052 (0.908, 1.220) | 0.500 |
| DN B cell at T6 | 1.010 (0.846, 1.207) | 0.909 |
| CD40 on naïve B cell at T0 | 0.124 (0, 43.742) | 0.485 |
| CD40 on naïve B cell at T1 | 79.523 (0, 56890000) | 0.525 |
| CD40 on naïve B cell at T3 | 0 (0, 943200) | 0.263 |
| CD40 on naïve B cell at T6 | 96.060 (0, 21580000) | 0.035 |
| CD80 on switched B cell at T0 | 0.976 (0.909, 1.047) | 0.495 |
| CD80 on switched B cell at T1 | 1.116 (0.900, 1.384) | 0.318 |
| CD80 on switched B cell at T3 | 1.013 (0.921, 1.114) | 0.788 |
| CD80 on switched B cell at T6 | 1.021 (0.938, 1.111) | 0.634 |
| CD95 on switched B cell at T0 | 0.976 (0.928, 1.027) | 0.352 |
| CD95 on switched B cell at T1 | 1.009 (0.935, 1.088) | 0.818 |
| CD95 on switched B cell at T3 | 1.118 (0.965, 1.296) | 0.137 |
| CD95 on switched B cell at T6 | 1.127 (0.954, 1.331) | 0.159 |
| CD21^low^ on DN B cell at T0 | 1.047 (0.965, 1.135) | 0.270 |
| CD21^low^ on DN B cell at T1 | 1.007 (0.932, 1.089) | 0.857 |
| CD21^low^ on DN B cell at T3 | 1.092 (0.938, 1.271) | 0.257 |
| CD21^low^ on DN B cell at T6 | 1.050 (0.951, 1.159) | 0.335 |
| CD22 on naïve B cell at T0 | 1.570 (0.297, 8.297) | 0.595 |
| CD22 on naïve B cell at T1 | 0.922 (0.383, 2.220) | 0.857 |
| CD22 on naïve B cell at T3 | 0.965 (0.723, 1.286) | 0.807 |
| CD22 on naïve B cell at T6 | 1.551 (0.473, 5.085) | 0.469 |
| p-SYK on non-switched B cell at T0 | 0.985 (0.004, 268.035) | 0.996 |
| p-SYK on non-switched B cell at T1 | 19.056 (0.001, 262500) | 0.544 |
| p-SYK on non-switched B cell at T3 | 0.031 (0, 7540) | 0.583 |
| p-SYK on non-switched B cell at T6 | 1.002 (0.999, 1.004) | 0.217 |
| p- AKT on non-switched B cell at T0 | 0 (0, 72.991) | 0.121 |
| p-AKT on non-switched B cell at T1 | 0.520 (0.213, 1.273) | 0.152 |
| p-AKT on non-switched B cell at T3 | 1.005 (0.968, 1.044) | 0.781 |
| p-AKT on non-switched B cell at T6 | 0.969 (0.001, 637.204) | 0.992 |

Abbreviations: CI, conﬁdence intervals; DN, double negative; OR, odds ratios; SLE, systemic lupus erythematosus; SLEDAI-2K, SLE Disease Activity Index 2000; T, time.
