# Supplementary figures and images for: The rapid inhibition of B-cell activation markers by belimumab was associated with disease control in systemic lupus erythematosus patients
Source: Front Pharmacol. 2023 Feb 16;14:1080730. doi: 10.3389/fphar.2023.1080730 (PMC9978353; doi:10.3389/fphar.2023.1080730)

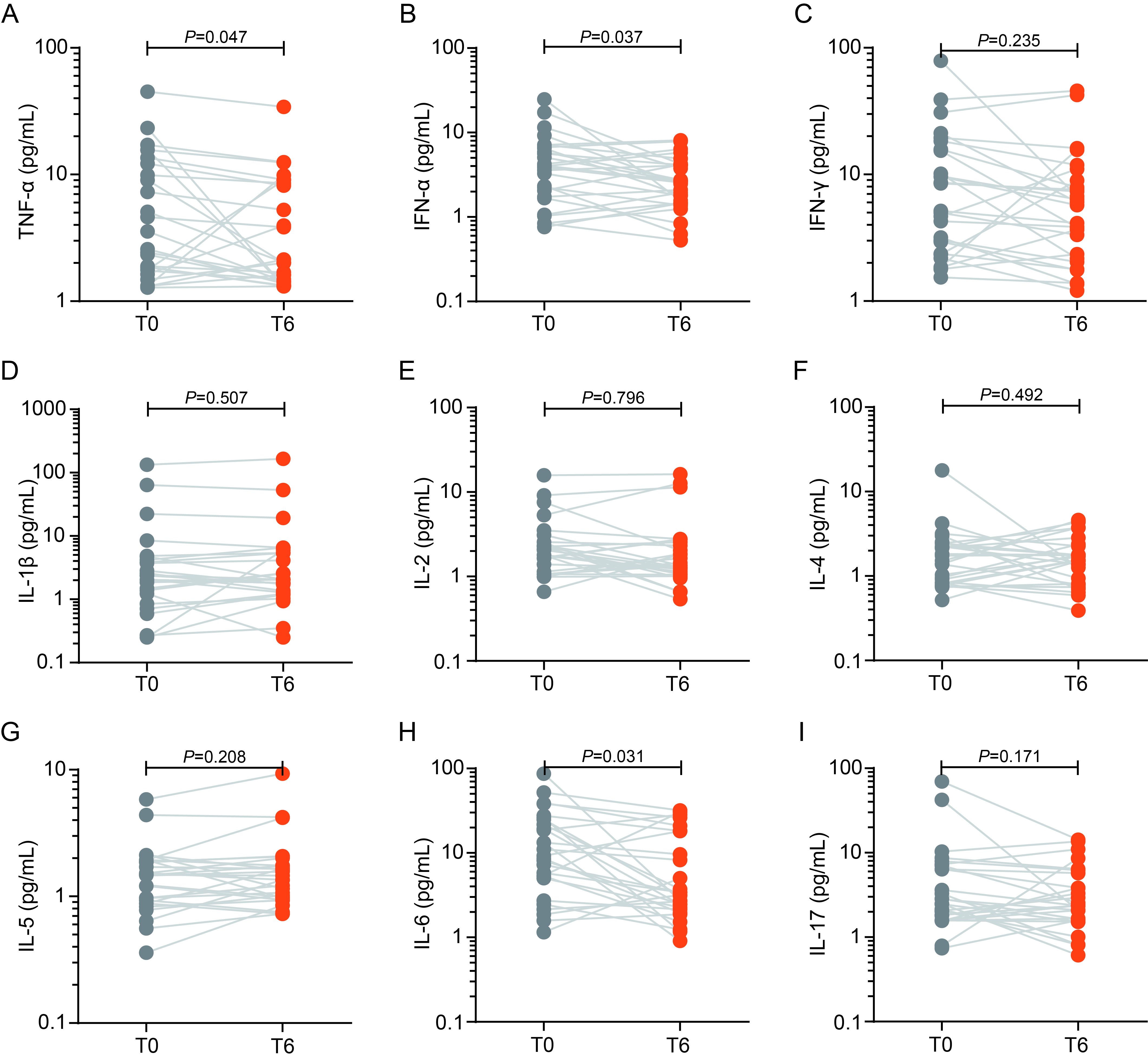

Supplement: Supplementary file 2 [file Image1.JPEG]
